# Effects of Lactobacillus* acidophilus* and *Bifidobacterium bifidum* probiotics on the serum biochemical parameters, and the vitamin D and leptin receptor genes on mice colon cancer

**DOI:** 10.22038/ijbms.2019.32624.7806

**Published:** 2019-06

**Authors:** Peyman Ranji, Shahram Agah, Zahra Heydari, Mohammad Rahmati-Yamchi, Ali Mohammad Alizadeh

**Affiliations:** 1 Cancer Research Center, Tehran University of Medical Sciences, Tehran, Iran; 2 Colorectal Research Center, Iran University of Medical Sciences, Tehran, Iran; 3 Drug Applied Research Center, Tabriz University of Medical Sciences, Tabriz, Iran; 4 Department of Medical Biotechnology, Faculty of Advanced Medical Sciences, Tabriz University of Medical sciences, Tabriz, Iran; 5 Cancer Biology Research Center, Tehran University of Medical Sciences, Tehran, Iran

**Keywords:** Colon cancer, Leptin receptor, Mice, Probiotic, Vitamin D receptor

## Abstract

**Objective(s):**

The preclinical reports have shown that specific probiotics like *Bifidobacterium bifidum* (*B. bifidum*) and *Lactobacillus acidophilus* (*L. acidophilus*) can be applied as the biotherapeutic agents in the inhibition or therapy of colorectal cancer via the modification of gut bacteria. In the previous studies, we have assessed the impact of *L. acidophilus* and *B. bifidum* probiotics on gut bacteria concentration and also their chemo-protective impact on mice colon cancer. In the following, we assessed the effects of these probiotics on the gene expression of vitamin D receptor (VDR) and the leptin receptor (LPR) and the serum biochemical parameters on mice colon cancer.

**Materials and Methods:**

Thirty-six male BALB/c mice were equally shared into 4 groups; (i) health with routine dietary foods without any treatment, (ii) azoxymethane (AOM)-induced mice colon cancer with common dietary foods, (iii) and (iv) AOM-induced mice colon cancer with oral consumption of *L. acidophilus* and *B. bifidum* (1×10^9^ cfu/g) for 5 months, respectively. Then, the serum total cholesterol, triglycerides, low-density lipoprotein cholesterol (LDL), high-density lipoprotein cholesterol (HDL), alanine transaminase, alkaline phosphatase, and albumin and also VDR and LPR genes expression were evaluated.

**Results:**

Oral consumption of *L. acidophilus* and *B. bifidum* probiotics significantly decreased the triglycerides, alkaline phosphatase, LDL, and also the VDR and LPR gene expression in mice colon cancer (*P<*0.005).

**Conclusion:**

*L. acidophilus* and *B. bifidum* probiotics with the modification of the biochemical parameters and the expression of the VDR and LPR genes can play a key role in the protection of mouse colon cancer.

## Introduction

A number of the reports have shown that the special probiotics such as *Bifidobacterium* and *Lactobacillus* can modify the gut bacteria that may be used as the biotherapeutic agents in the inhibition or therapy of colorectal cancer (CRC) (1-3). In the prior experiment, we assessed the impact of *Lactobacillus acidophilus (L. acidophilus)* and *Bifidobacterium bifidum (B. bifidum)* probiotics on the concentration of intestinal bacteria. In this context, the concentration of *L. acidophilus* remained almost unchanged during 5 months and showed a more stability compared to *B. bifidum* probiotic (4). We have also compared the role of *L. acidophilus* with *B. bifidum* probiotic on the azoxymethane (AOM)-induced mice colon cancer. In this regard, *L. acidophilus* probiotic consumption with a significant increase of the serum levels of interferon-γ (IFγ) and interleukin 10 (IL-10), and also the CD8+ and CD4+ cells could have a more antitumor activity than *B. bifidum* probiotic. Therefore, the replacement of the pathogenic bacteria by the probiotics can be a potential mechanism of the immune system responses that may modulate colon cancer severity (5).

During immune responses, the studies have demonstrated that the vitamin D receptor (VDR) activation up-regulate the expression of cathelicidin and defensin that can control the concentration of the bacterial flora, though the function of the vitamin D/VDR in regulating bacterial flora remains unidentified (1). Another study demonstrated that vitamin D3 can activate the VDR to inhibit the tumor cell development by a differentiation induction in the various tumor cells such as the intestinal cancer cells (6-8). Moreover, the recent studies have shown that the VDR−/− mice can increase the bacterial concentration in the gut and also the induction of both commensal and pathogenic bacteria (9, 10). In this context, the probiotics can modulate the anti-inflammatory signaling pathway of the VDR in colitis with an unknown mechanism. Furthermore, there is the growing evidence that the VDR can control the function of the leptin and other class I cytokines (11, 12). Leptin has been reported that can decline the renal 25-hydroxyvitamin D (3)-1α-hydroxylase expression in mice by binding to the leptin receptor (LPR) in colon cancerous cells (19). LPR is a kind of the pro-inflammatory cytokine receptor and its high circulating level has been observed in the advanced stage of CRC patients (13, 14). Although some clinical studies reported the low level of the serum leptin in CRC patients, the high serum leptin level was associated with the high incidence of colon cancer in other studies (15-18). Thus, it seems that the probiotics with an improvement of the microflora bacteria concentration can have beneficial effects on the LPR and VDR gene expression. In this respect, the present study was aimed to assess *L. acidophilus* and *B. bifidum* impacts on the VDR and LPR gene and the serum biochemical parameters on mouse colon cancer. 

## Materials and Methods

***Materials***

*B. bifidum* and *L. acidophilus* were gifted from Zist Takhmir Supplements Company (Tehran, Iran). Ketamine, xylazine, and AOM were provided from Sigma Aldrich Co. (St Louis, MO). Geneall ^R ^Hybrid-R^TM^-miRNA Kit (Cat.No:325-150) and M-MuLV Reverse Transcriptase (Cat. No: PR911658) (10000 units) from CinaGen Company (Tehran, Iran) were used for extracting RNA, and DNA synthesis, respectively. 5x HOT FIREPol EvaGreen qPCR Mix Plus (no ROX) were purchased from Solis BioDyne (Tehran, Iran). Biosino Biotech and Sci, Inc, Beijing, China Elisa kits were used for assessment of the biochemical parameters. 

***Animals***

Thirty-six male BALB/c mice (6-8 weeks old) were bought from Pasteur Institute of Iran and housed in the high groups under 12-hr day/night cycles. The procedures were in the agreement with Tehran University of Medical Sciences instructions for the maintenance and use of the laboratory animals and the ethical standards of the institutional and/or national research committee and with the 1964 Declaration of Helsinki and its last amendments ethical standards.

***Study design***

According to the study protocol, the animals were equally divided into 4 groups; (i) the health with ordinary dietary foods without any treatment, (ii) the AOM-induced colon cancer, (iii) *L*. *acidophilus,* and (iv) *B.* *bifidum*. The study design and protocol were the same as our previous work (5). In the II-IV groups, AOM (15 mg/kg, subcutaneous (SC)) was weekly injected in three continues weeks to create mouse colon cancer. Oral consumption with *L*. *acidophilus *(1×10^9 ^cfu/g Lac/002P/M) in the iii group and the nutrition with *B.* *bifidum* (1×10^9 ^cfu/g Bla/016P/M) in the iv group were started ten days before the AOM injection and daily continued sustained for 5 months (5, 19)

***Blood and tissue sampling***

At the end of the 20^th^ week, the mice were finally euthanized using a cervical dislocation under the general anesthesia with the combination of the ketamine and xylazine (10:1) at a 110 mg/kg body weight dose by a SC injection. Almost 1.5 ml of the blood samples were collected and subsequently centrifuged for ten min at 3,000 rpm. Prepared serum was kept at −80 ^°^C for future examinations. Distal colon specimens were also excised and gently removed under a routine surgery for the RNA extraction.

***Hematology and blood chemistry tests***

At the end of the treatment, we euthanized the mice under the general anesthesia. Blood samples were isolated and stored into the coated tubes by ethylene-diamine-tetra-acetic-acid (EDTA) for haematology, and the coated heparin for the clinical chemistry. The serum total cholesterol (TC), triglycerides (TG), low-density lipoprotein cholesterol (LDL-C) and high-density lipoprotein cholesterol (HDL-C), alanine transaminase (ALT), alkaline phosphatase (ALP), and albumin (ALB) were enzymatically measured with an Autoanalyzer System (Autoanalyser Model Biotecnica, BT 3500, Rome, Italy) and a commercial kit (Biosino Biotech & Sci, Inc, Beijing, China) (20, 21). 

***RNA-extraction and real-time PCR***

Total RNA was extracted from the tissue of colon tumors with Gene all Hybrid-R miRNA kit (Cat No: 325-150, Lot. No: M 3250 19L) based on the manufacturer’s guidelines. The quantity and quality of the extracted RNAs were measured by Thermo Scientific Nanodrop 2000 C Spectrophotometer (USA) and an agarose gel electrophoresis (1% agarose; Gibco/BRL), respectively. The amount of the 260/280 OD ratio of all samples was confirmed between 1.8 and 2.2, showing their high purity. cDNAs were synthesized by a M-MuLV Reverse Transcriptase cDNA Synthesis Kit (10000 units) from CinaGen company (Tehran, Iran) according to the manufacturer’s instruction, and kept at −20 ^°^C. Primers were designed in two adjacent exons by Shangaye Generay Biotech (Table 1). All of the selected primer sequences were checked with the Oligo software and the primer blast in the NCBI database. The level of the mRNA expression was normalized by using beta-actin as an internal housekeeping control gene. Real-time PCR was carried out by a light cycler tool (Applied Biosystems 7500, USA) and 5x HOT FIREPol^®^ EvaGreen®qPCR Mix Plus (no ROX) (Solis BioDyne Inc). In a total volume of 20 μl, 14 μl of nuclease-free water, 4 μl Eva Green master mix, 1 μl of cDNA samples as well as 0.5 μl of forward and 0.5 μl reverse primers (Qiagen, Hilden, Germany) were transferred into each capillary tube. The PCR condition was involved an initial denaturation of 15 min at 95 ^°^C, 40 cycles at 95 ^°^C for 15 sec, 62 ^°^C for 20 sec and 72 ^°^C for 20 sec, respectively. The specificity of the PCR products was evaluated by confirming a single peak in the melting curve analysis. The stained 1.5% agarose gel with ethidium bromide were used for a complementary length verification (22). 

***Statistical analysis***

Comparison among groups was determined by the analysis of variance (ANOVA) and Tukey tests. Chi-square test was also used for a ratio comparison. Statistical significance was defined by mean±SD. Statistical analysis was performed applying the SPSS statistical software version 20.

## Results

***Biochemical assay and the serum level of the lipid profile***

We have measured the serum level of the lipid profile including TC, HDL-C, LDL-C, and TG with the ELISA method. We have found that the LDL-C and TG were significantly increased in the AOM group (34.0±8 and 158±49 mg/dl, respectively) in comparison with the health group (9.7±5.8 mg/dl for LDL and 86±12 mg/dl for TG) (*P˂0.05*). Our results have also demonstrated a considerable reduction of the LDL-C and TG in *L. acidophilus* (7.0±3.6 and 91±23 mg/dl, respectively) and *B. bifidum* (12±13 and 90±13 mg/dl, respectively) groups in comparison with the AOM group (34.0±8 and 158±49 mg/dl, respectively) (*P˂0.05*). Compared to the health group (70±6 mg/dl), the HDL was non-significantly decreased in the AOM group (53±23 mg/dl). However, in comparison with the AOM group (53±23 mg/dl), a significant and non-significant increase of the HDL serum level was seen in *L. acidophilus *(72±15 mg/dl) and *B. bifidum* (63±11 mg/dl) groups, respectively. Furthermore, *B. bifidum* non-significantly declined the TC (90±43 mg/dl) in comparison with the AOM group (103±39 mg/dl), but *L. acidophilus *did not demonstrate a considerable change in the TC (Table 2).

Measurements of the other biochemical markers including the ALP, ALT, and ALB were performed in our study. In this regard, oral consumption of *L. acidophilus* significantly and non-significantly decreased the ALP (143±50 U/L) and ALT (90±14 U/L) in contrast to the AOM group (200±33 U/L for the ALP and 102±26 U/L for the ALT), respectively (*P˂0.05*). Moreover, we found that *B. bifidum* non-significantly decreased the ALP (181±11 U/L) and ALT (89±17 U/L) versus the AOM group. In spite of the ALP and ALT, a considerable change of the ALB was not seen in *L. acidophilus* and *B. bifidum* groups compared to the AOM group (Table 2).

**Figure 1:**
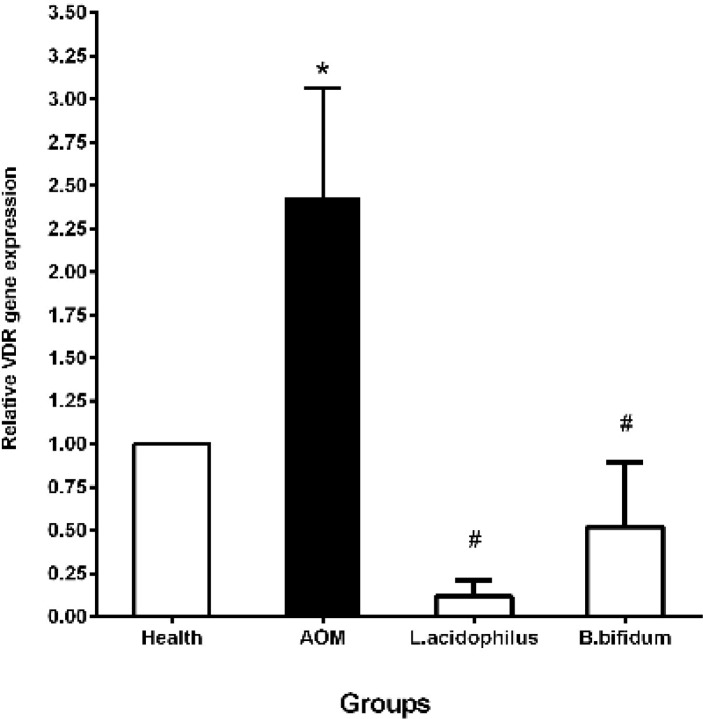
Effects of *L. acidophilus* and *B. bifidum* probiotics on the VDR gene expression in a mouse model of colon cancer

Data reported are mean±SD; *P<0.05 compared to the health group; #*P<*0.05 compared to the AOM group; $*P<*0.05 compared to the *B. bifidum*; VDR=vitamin D receptor; AOM: azoxymethane, *B. bifidum: Bifidobacterium bifidum, L. acidophilus: Lactobacillus acidophilus: *

**Figure 2:**
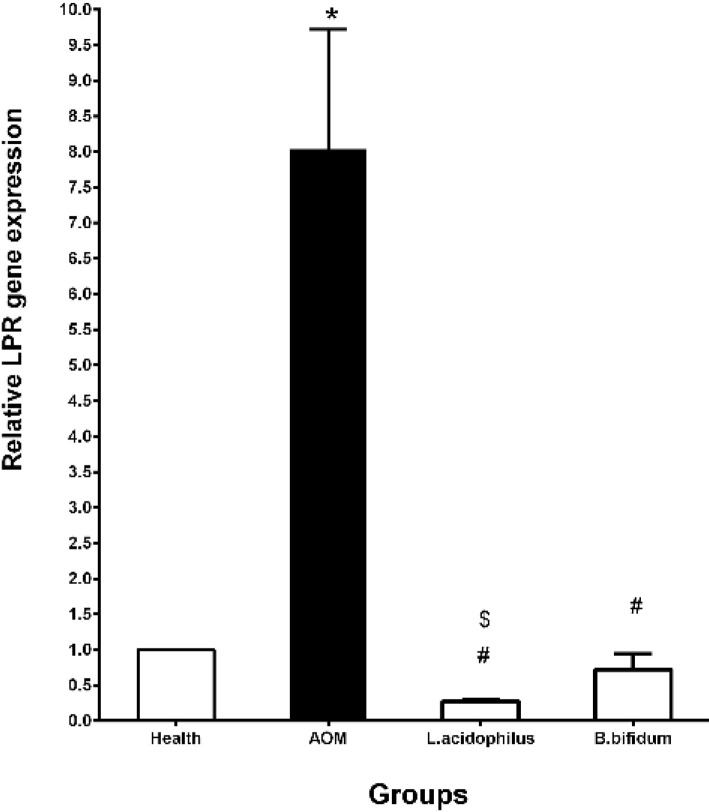
Effects of *L. acidophilus* and *B. bifidum* probiotics on the LPR gene expression in a mouse model of colon cancer

Data reported are mean±SD; *P<0.05 compared to the health group; #*P<*0.05 compared to the AOM group; $*P<*0.05 compared to the *B. bifidum* group; LPR= Leptin receptor; AOM: azoxymethane, *B. bifidum: Bifidobacterium bifidum, Lactobacillus acidophilus: L. acidophilus*

**Table 1:** The sequences of the specific primers for the β-actin, LPR, and VDR

**Gene name**	**Reverse primer**	**Forward primer**
VDR	5’-TGATCGTGTTCTCTCCCAGTTC-3’	5’-AGAAGTGCCCAAAGGGAAGTC-3’
LPR	5’-GCTCCATTCTTGATCCCAGAG-3’	5’-TAGACCAGCAGTGGCTTGTTG-3’
β-actin	5’-CCAGTTGGTAACAATGCCATGT-3’	5’-GGCTGGTATTCCCCTCCATCG-3’

VDR: Vitamin D receptor; LPR: Leptin receptor

**Table 2:** Effects of *Lactobacillus acidophilus *and *Bifidobacterium bifidum* probiotics on the biochemical parameters in comparison with the AOM and health groups in a mouse model of colon cancer

Groups	**Health**	**AOM**	***L. acidophilus***	***B. bifidum***
Parameters				
**Cholesterol (mg/dl)**	107 ± 32	103 ± 39	102 ± 43	90 ± 43
**TG (mg/dl)**	86 ± 12	158 ± 49 *	91 ± 23 #	90 ± 13 #
**HDL (mg/dl)**	70 ± 6	53 ± 23 *	72 ± 15 #	63 ± 11
**LDL (mg/dl)**	9.7 ± 5.8	34 ± 8 *	7 ± 3.6 #	12 ± 13 #
**ALP (U/L)**	128 ± 20	200 ± 33 *	143 ± 50 # $	181 ± 11
**ALT (U/L)**	64 ± 7.8	102 ± 26 *	90 ± 14	89 ± 17
**ALB (g/dl)**	2.8 ± 0.14	2.9 ± 0.14	3.1 ± 0.1	2.9 ± 0.2

Data reported are mean ± SD;

HDL: High-density lipoprotein; LDL: low-density lipoprotein; TG: triglycerides; ALT: alanine transaminase; ALP: alkaline phosphatase; ALB: serum albumin; AOM: azoxymethane; *Lactobacillus acidophilus*: *L. acidophilus****;*** *Bifidobacterium bifidum*: *B. bifidum.*

* *P<*0.05 compared to the health group;

# *P*<0.05 compared to the AOM group;

$ *P<*0.05 compared to *the B. bifidum.*

***Gene expression patterns***

The expression of the VDR and LPR genes was evaluated in each of 4 groups with the real-time PCR method. Results in the present study indicated that the probiotics decreased the VDR and LPR gene expressions in comparison with the AOM and health groups. In this context, the VDR gene expression was significantly decreased in *B. bifidum* (0.52±0.37) and *L. acidophilus* (0.12±0.09) groups in comparison with the AOM group (2.43±0.63) (*P*˂0.05). Compared to the health group, the expression of the VDR gene was considerably increased in the AOM group (2.43±0.63) (*P*˂0.05) (Figure 1). Moreover, we found that *L. acidophilus* (0.27±0.03) and *B. bifidum* (0.72±0.22) probiotics significantly decreased the expression of the LPR gene against the AOM group (8.04±1.68) and the healthy group after 20 weeks. Similar to the VDR expression pattern, the LPR gene expression was significantly gone up in the AOM group (8.04±1.68) compared to the health group (Figure 2). Likewise, *L. acidophilus* (0.27±0.03) considerably declined the LPR gene expression versus *B. bifidum* (0.72±0.22) probiotic (Figure 2).

## Discussion

The major aim of the current study was to assess *L. acidophilus* and *B. bifidum *effects on the serum biochemical parameters, and the VDR and LPR genes in mice colon cancer. Our present study results have demonstrated that *L. acidophilus* and *B. bifidum* significantly decreased the serum level of the LDL and TG, and the gene expression of the LPR and VDR in comparison with the AOM group. Unlike *B. bifidum*, *L. acidophilus* considerably increased the serum level of the HDL in comparison with the AOM group. Furthermore, the oral consumption of *L. acidophilus* and *B. bifidum* significantly and non-significantly decreased the ALP, respectively. Thus, an oral consumption of *L. acidophilus* and *B. bifidum* probiotics could be effective on the serum biochemical parameters, and the VDR and LPR genes in mice colon cancer. 

The data collected from *in vitro* studies were verified the anti-cancer effects of the *Lactobacillus* probiotic in CRC studies through various pathways. For instance, *Lactobacillus* strains have the ability to inhibit the growth of the HT-29 cells via the Bax/Bcl-2 pathway or NO production (23). Moreover, the exopolysaccharides from nine *Lactobacillus* strains have shown the inhibitory effects on the HT-29 cells via an induction of the apoptosis and up-regulation of the caspase-3 activity (24). Furthermore, *L. acidophilus* exopolysaccharides represented the anti-cancer potentialities against the CaCo-2 cells through the apoptotic and NF-κB inflammatory pathways (25). Moreover, our previous study indicated that unlike *B. bifidum*, the amount of *L. acidophilus* did not have any change in the mice colon cancer for 5 months (4). We have also concluded that the stability of *L. acidophilus* in the microflora concentration has the beneficial effects in the decrease of the CRC development with a significant decrease of the incidence and size of the colon tumor, and CA19-9 and CEA markers (5). The collected data from the previous studies have also demonstrated that there is a positive correlation between the serum lipids including the TC, LDL-C, and TG with a risk of the CRC (26). The current results have indicated that 20 weeks oral consumption of *L. acidophilus* and *B. bifidum* could meaningfully decrease the LDL-C and TG compared to the AOM group. Moreover, *L. acidophilus* with a significant increase of the HDL serum level has a more beneficial effect than *B. bifidum*. In this regard, the supplementation of *L. acidophilus* in combination with other probiotics and phytosterols could decrease the TC and LDL-C in hypercholesterolemic rats (27). In addition, yogurt containing *L. acidophilus* and *B. lactis* probiotics has dropped the TC and LDL-C concentrations in the type 2 diabetes patients (28). We have also assessed the other biochemical parameters such as the ALP and ALT that have been shown to increase about 30% of the patients with the liver metastases of the CRC (10). In this context, our study results have also indicated that *L. acidophilus* has significantly declined the ALP as compared to the *B. bifidum* and the AOM groups. An i*n vitro* study demonstrated that the incubation of *Lactobacillus rhamnosus GG* in the Caco-2 cells caused the non-significant change in the ALP activity (29). Moreover, the consumption of *Bifidobacterium longum* and *L. acidophilus* indicated no effect on the ALP level of the fecal sample in the injected rats with 1, 2-dimethylhydrazine dihydrochloride (30). In contrast, the administration of *Bifidobacterium spp* decreased the TC, HDL-C, LDL-C, TG, leptin, and ALT in the high-fat diet-induced obese rats (31). Therefore, it seems that a reduction of the TC, LDL-C, TG, and ALP with *L. acidophilus* and *B. bifidum* probiotics might be a method for the protection of the CRC (26, 32). 

Our results also demonstrated that the VDR gene expression was considerably climbed in the AOM group in comparison with the health group. In the preclinical studies, the contradictory outcomes have observed the VDR gene expression in colon cancer models. In this context, a high-level expression of the VDR has been observed in an early colorectal tumor progression, but a loss of the VDR expression was reported during the tumor dedifferentiation. Moreover, a high VDR expression was observed in the proximal colon compared to the distal colon, which can be correlated with the gut bacterial concentration (1). In our study, *L. acidophilus* and *B. bifidum* probiotics significantly declined the VDR gene expression in comparison with the AOM group. Treatment with the VSL (a probiotic including eight different strains of bacteria) has meaningfully raised the VDR expression in the proximal and distal colons in a rat model of colitis-associated cancer (33). This contradictory in results may be due to the difference in the type of animal models and/or the probiotic strains. On the other hand, there are associations between the VDR expression and the gut bacterial concentration. Thus, the probiotics with the modulation of the gut bacterial concentration can have useful effects on the VDR gene expression. However, the biological mechanisms between the VDR functions and gut bacteria are unclear and remain a main challenge in the future. 

Similar to the VDR expression pattern, we have found that the LPR gene expression has significantly decreased in the AOM group versus the health group. A high level of the LPR expression was observed in the AOM group (34). Moreover, the results of other studies have indicated that the expression of the leptin and its receptor in the CRC patients could correlate with the tumor differentiation grade and depth of bowel wall invasion (13, 35). Furthermore, the results of a clinical study in the United States demonstrated that there is a relationship between the concentration of the microflora bacteria and the LP/LPR (36). Moreover, a preclinical study has shown that the VSL could decline the leptin expression in the mesenteric adipose tissue in the Crohn’s patients  (37). In the present study, we have also found that 20 weeks oral consumption of *L. acidophilus* and *B. bifidum* probiotics considerably decreased the LPR gene expression in the mouse model of the AOM-induced colon cancer. Thus, *L. acidophilus* and *B. bifidum *probiotics with an improvement of the microflora bacteria have a decreasing effect on the LPR gene expression.

## Conclusion

The concentration of the microflora bacteria, leptin/LPR, and vitamin D/VDR has been associated with colon cancer as environmental factors. Moreover, the previous studies displayed that the high serum level of the LDL-C and TG and also the high activity of the ALP are correlated with the CRC. Our results in the present study demonstrated that *L. acidophilus* and *B. bifidum* have significantly decreased the LDL-C, TG, LPR, and VDR gene expression compared to the AOM group. It seems that *L. acidophilus* with a significant increase in the HDL serum level and a significant decrease in the ALP activity has a more beneficial effect than *B. bifidum *in mouse colon cancer. Thus, *L. acidophilus* and *B. bifidum* probiotics with the modification of the microflora bacteria concentration and their effect on the LPR, VDR, and the mentioned biochemical parameters may play an essential role on the CRC. However, the biological mechanisms between the mentioned options and gut bacteria are unclear, and future evaluations are necessary for defining an exact mechanism.
